# The *CARPEDIEM* Algorithm: A Rule-Based System for Identifying Heart Failure Phenotype with a Precision Public Health Approach

**DOI:** 10.3389/fpubh.2018.00006

**Published:** 2018-01-29

**Authors:** Michela Franchini, Stefania Pieroni, Claudio Passino, Michele Emdin, Sabrina Molinaro

**Affiliations:** ^1^Institute of Clinical Physiology, National Research Council, Pisa, Italy; ^2^Division of Cardiology and Cardiovascular Medicine, Fondazione Toscana Gabriele Monasterio, Pisa, Italy; ^3^Institute of Life Sciences, Scuola Superiore Sant’Anna, Pisa, Italy

**Keywords:** algorithm, phenotype, precision public health, heart failure, accuracy, predictive measures

## Abstract

Modern medicine remains dependent on the accurate evaluation of a patient’s health state, recognizing that disease is a process that evolves over time and interacts with many factors unique to that patient. The *CARPEDIEM* project represents a concrete attempt to address these issues by developing reproducible algorithms to support the accuracy in detection of complex diseases. This study aims to establish and validate the *CARPEDIEM* approach and algorithm for identifying those patients presenting with or at risk of heart failure (HF) by studying 153,393 subjects in Italy, based on patient information flow databases and is not reliant on the electronic health record to accomplish its goals. The resulting algorithm has been validated in a two-stage process, comparing predicted results with (1) HF diagnosis as identified by general practitioners (GPs) among the reference cohort and (2) HF diagnosis as identified by cardiologists within a randomly sampled subpopulation of 389 patients. The sources of data used to detect HF cases are numerous and were standardized for this study. The accuracy and the predictive values of the algorithm with respect to the GPs and the clinical standards are highly consistent with those from previous studies. In particular, the algorithm is more efficient in detecting the more severe cases of HF according to the GPs’ validation (specificity increases according to the number of comorbidities) and external validation (NYHA: II–IV; HF severity index: 2, 3). Positive and negative predictive values reveal that the *CARPEDIEM* algorithm is most consistent with clinical evaluation performed in the specialist setting, while it presents a greater ability to rule out false-negative HF cases within the GP practice, probably as a consequence of the different HF prevalence in the two different care settings. Further development includes analyzing the clinical features of false-positive and -negative predictions, to explore the natural clustering of markers of chronic conditions by adding additional methodologies, e.g., Social Network Analysis. *CARPEDIEM* establishes the potential that an algorithmic approach, based on integrating administrative data with other public data sources, can enable the development of low cost and high value population-based evaluations for improving public health and impacting public health policies.

## Introduction

In today’s healthcare environment, both physicians and patients are the beneficiaries of great scientific and technologic advances. However, modern medicine remains dependent on the accurate evaluation of a patient’s health state, recognizing that disease is a process that evolves over time and interacts with many factors unique to that patient. As a result, the most critical factors that must be determined are as follows: (1) what is the disease path/process that a patient is exhibiting, i.e., the diagnosis, and (2) how far along that path has the patient progressed, i.e., disease staging. The entire premise of precision medicine requires the ability to determine both diagnosis and disease stage with a high degree of accuracy so that the appropriate selection of treatment pathway can be applied to optimize outcome (1).

In spite of the increased access to clinical data resources, there remains a lack of standardization of definitions and formats that negatively affects interoperability of health records. This further limits the development of comprehensive and universally adopted standards for diagnosis and treatment using evidence-based medicine and reduces the accuracy in establishing a patient’s true health condition. The US Institute of Medicine estimates that approximately 10% of all diagnoses are incorrect. This estimate includes both type 1 errors (false-positive diagnoses) and type 2 errors (false-negative diagnoses) and leads to unnecessary patient deaths (estimated at 40,000–80,000 per year in the United States) with an estimated cost of $750 billion per year in the United States. Death due to medical errors has been estimated to be between 200,000 and 400,000 per year (the United States) and places this cause just behind heart disease and cancer with misdiagnosis and missed diagnosis representing between 15 and 25% of all medical errors (2). In addition, these numbers significantly underestimate healthcare costs associated with non-fatal misdiagnoses and missed diagnoses that result in inappropriate but non-fatal treatment of patients (3).

Heart failure (HF) is one of the most important public health problems encountered in the developed world. Despite the introduction of new treatments, HF is associated with high costs and poor outcomes. Many of the patients exhibit non-specific symptoms, which makes it difficult to identify HF and distinguish it from other conditions. Thus, many patients may have undiagnosed HF, or even when diagnosed, other undiagnosed concomitant conditions, as diabetes that is common in patients with acute HF, may confound the HF diagnosis. It is important to identify these patients and provide access to appropriate treatment to reduce mortality, improve healthcare, and reduce costs derived from undiagnosed disease (4, 5).

The development of rule-based systems for organizing and presenting information extracted from multiple sources of standardized data related to large patient populations that facilitate problem solving and decision making in the clinical field is the first step toward establishing Precision Public Health (6).

In Italy, common and interoperable syntaxes have been developed for the National Health System (NHS) subcomponents (hospitalization, outpatient specialized treatment, and others). These enable collection, in specific data flows, of all information (individual characteristics, treatments/drugs, tariffs, co-pay fee exemption) generated when citizens interact with the NHS. The use of these standardized information models facilitates their integration from different data sources. This enables the development of more precise diagnostic criteria; moreover performing the analysis on large databases that include many different healthcare processes and events, enhances patient phenotype identification/assignment.

Although the primary purpose of these sources of data is to collect, store, and exchange patient information to allow administrative management of the NHS (7), in this manner, they may be also used to identify patient cohorts by first deriving rule-based phenotyping from the combination of standards, archetypes, ontology, and reasoning (8) and then application of these algorithms to screen all patients in the database for potential candidates with as yet undiagnosed disease.

This effort to develop phenotyping algorithms requires consistency in data (archetypes level) and knowledge level (ontology) simultaneously because diagnostic criteria (reasoning) are calculated from raw data but also need derived variables not directly available in original data (8, 9).

Furthermore, administrative data represent not only a patients’ clinical characteristics but also the manner in which information is collected and recorded during the healthcare process events, ranging from inpatient admission, inpatient discharge, outpatient visit, emergency department visit, and ambulatory surgery (10). The propensity of the variables for clustering based on their association with specific healthcare process events results in better derivation of phenotyping algorithms for cohort/classification (10).

This study was undertaken to establish and validate the *CARPEDIEM* approach and algorithm for identifying those patients presenting with or at risk for HF to streamline the development of patient registries for supporting the proactive approach inspired by the Chronic Care Model, developed by Wagner in the late 1990s (11). This model assumes that improvement in care requires an approach that incorporates patient, provider, and system level interventions and that better coordination of care may also reduce medical expenditures, especially for persons with multiple chronic conditions (12).

## Materials and Methods

### Reference Cohort and Data Sources

The reference cohort (153,393 subjects) used in this study includes all patients older than 14 years attending those general practitioners (GPs) who participated in the PHP model in 2008–2012 in two different geographical areas in the center of Tuscany (area 1: 83 GPs; area 2: 31 GPs).

The administrative data used to detect HF cases include (1) an archive of all residents in the Tuscany areas receiving NHS assistance storing demographic and administrative data, including the GP’s identifier of each subject, (2) hospital discharge forms from public and/or private hospitals reporting all diagnoses related to hospitalizations, (3) all outpatient drug prescriptions reimbursable by the NHS, (4) certifications of chronic disease for the exemption from co-payment, and (5) all outpatient prescriptions for visits, laboratory/imaging tests, and medications.

Moreover, the pathology register that contains reporting from GPs about all their patients presenting HF was used as one of the standards for validating the algorithm. Apart from the last source of data referring to the period 2010–2012, the others referred to the period 2011–2012.

In all the archives, each patient’s record has been deidentified using an encrypted unique identifier code, which became the key element to link the sources of data through a deterministic approach.

Each final patient record, created through the linkage procedure, contained information about gender, age class, the presence or absence of comorbidities (the clinical features), and HF diagnosis as identified by GPs.

### The CARPEDIEM Algorithm Definition Process

**Figure 1:**
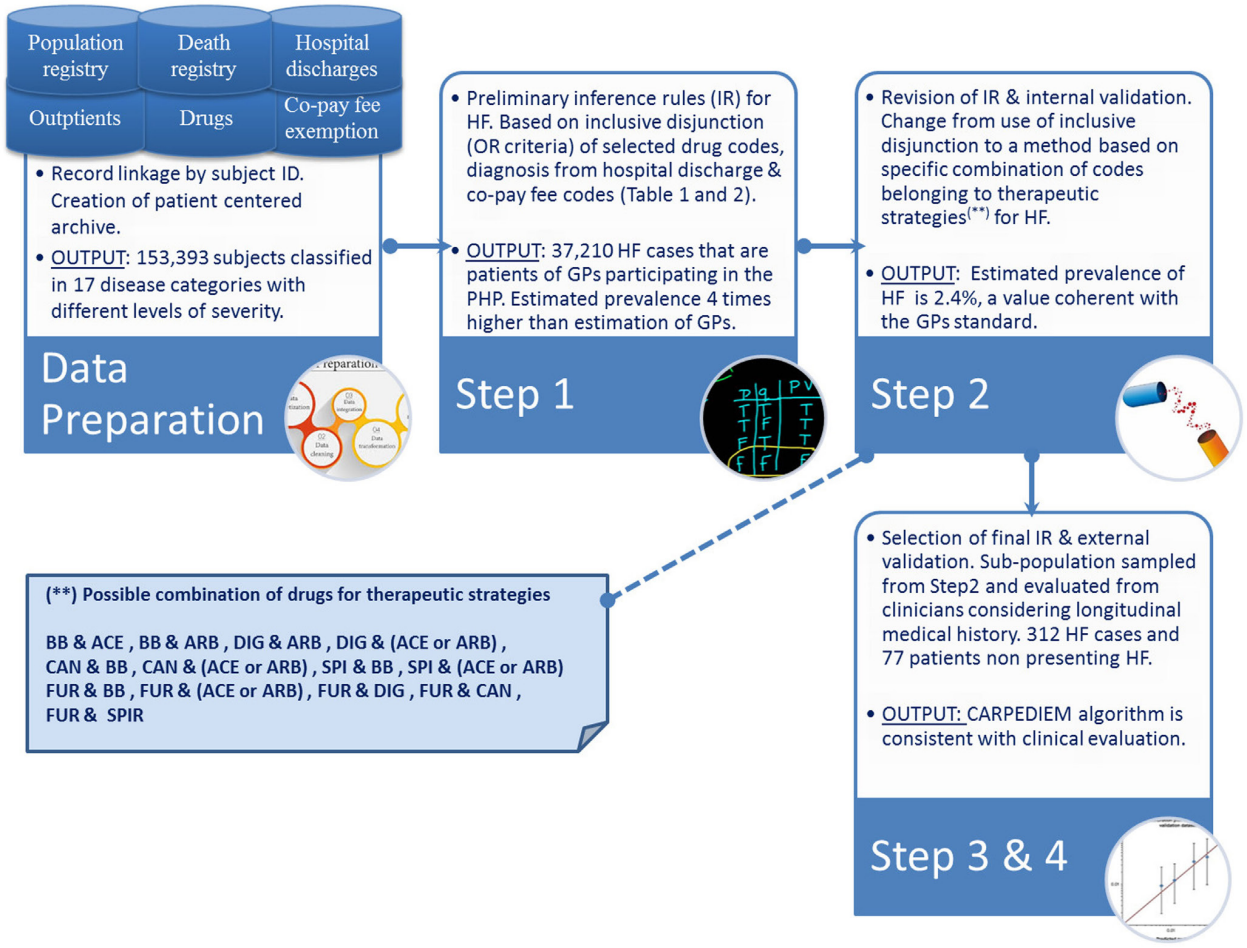
Schema of the *CARPEDIEM* algorithm.

The *CARPEDIEM* algorithm involves a four-step process (Figure 1), which includes (1) development of preliminary inference rules to build up the phenotype definition, (2) evaluation and revision of these rules, (3) selection of the final rules, and (4) subsequent validation by checking the rules against the standards.

The preliminary set of inference rules were developed by referring to a classification method that defines for each patient the groups of conditions to which he/she belongs. Through this model, each patient is classified based on criteria that consider (1) “exemption from co-payment codes” (codes elaborated by Italian Law M.D. of May 28, 1999 nr. 329 and further modifications), (2) diagnosis codes [International Classification of Diseases, Ninth Revision, Clinical Modification (ICD9-CM)] from hospital discharge, (3) drug classes as administered to the patient, and (4) related daily defined dose. Drug classes were defined using the Anatomical Therapeutic Chemical classification index derived from drug prescription data flow. The inclusion criteria were originally developed by the University of Pavia (13). The Pavia model defines 17 categories each with a different level of severity: the inference rules that define the 16 classifications other than “cardiovascular” were used to estimate the number and the type of patient’s comorbidities (14).

The preliminary inference rules identify an HF case if (1) he/she has drug prescriptions of β-blocker, spironolactone (SPI), furosemide (FUR), angiotensin-converting enzyme (ACE) inhibitors, angiotensin receptor blocker (ARB), digoxin (DIG), and canrenone as detailed in Table 1, (2) he/she has a “exemption from co-payment code” for HF (code 021), or (3) he/she has diagnosis of HF at hospital discharge, i.e., diagnosis of hypertensive cardiopathy with heart failure, cardiovascular hypertension with heart failure and chronic renal disease, cardiomyopathy, and heart failure (ICD9-CM codes listed in the Table 2).

**Table 1:** Drug codes included in the *CARPEDIEM* algorithm as markers of heart failure.

	Drug	Anatomical therapeutic chemical code
β-blocker	Bisoprolol	C07AB07
	Nebivolol	C07AB12
	Carvedilol	C07AG02
	Metoprolol	C07AB02

Angiotensin-converting enzyme inhibitors	Enalapril	C09AA02
	Captopril	C09AA01
	Lisinopril	C09AA03
	Ramipril	C09AA05

Angiotensin receptor blocker	Candesartan	C09CA06
	Losartan	C09CA01
	Valsartan	C09CA03

Other	Digoxin	C01AA05
	Canrenone	C03DA03
	Spironolacton	C03DA01
	Furosemide	C03CA01

**Table 2:** ICDIX codes included in the *CARPEDIEM* algorithm as markers of HF.

Diagnosis	ICD9-CM code
Hypertensive cardiopathy with heart failure	40201
	40211
	40291

Cardiovascular hypertension with heart failure and chronic renal disease	40401
	40403
	40411
	40413

Cardionephropathy hypertension with heart failure	40491
	40493

Cardiomyopathy	4254
	4255
	4257
	4258
	4259

Heart failure	4280
	4281
	4289

In general, the validity of rule-based identification system is assessed by comparing the diagnosis from the administrative database to an accepted “gold standard” reference diagnosis and by estimating the principal measures of validity such as sensitivity, specificity, predictive values, likelihood ratios (LRs), and Kappa scores.

Heart failure patients with clinical evidence documented by GPs represented the initial gold standard against which we tested the preliminary rules.

Heart failure prevalence rate estimated by the preliminary rules was considered as an indication of the false-positive rate and accuracy of the preliminary rules.

To improve the accuracy of the rule-based system, the criteria derived from semistructured individual and independent interviews with three cardiologists and from clinical guidelines were combined into a matrix of possible therapeutic strategies for HF, which might be identified from the administrative data.

In particular, during the interviews, the clinicians were asked to indicate which drug codes they thought should be grouped into combinations that are considered to be clinically appropriate for treating HF in its various levels of severity. The resulting grouping rules consider β-blockers (BBs), ACE inhibitor, ARBs, DIG, Candesartan (CAN), SPI, FUR with different possible combinations: (1) BB and ACE, (2) BB and ARB, (3) DIG and BB, (4) DIG and (ACE or ARB), (5) CAN and BB, (6) CAN and (ACE or ARB), (7) SPI and BB, (8) SPI and (ACE or ARB), (9) FUR and BB, (10) FUR and (ACE or ARB), (11) FUR and DIG, (12) FUR and CAN, and (13) FUR and SPI. Within a single combination, drug codes were taken in logical conjunction (AND connective), and then each drug combination was taken in inclusive disjunction (OR connective) with diagnoses (Table 2) and exemption from co-payment code for HF.

This leads to identifying a narrow rule-based system, the *CARPEDIEM* algorithm, which was tested both against the GPs and the clinical standard.

The external clinical validation was performed in a subpopulation of 389 patients (area 1: 269; area 2: 120) randomly sampled among those patients of the reference cohort who attended the Gabriele Monasterio Foundation (GM Foundation) for hospitalizations or outpatient activities during the observational period (2011–2014).

Those patients were clinically characterized through specific laboratory parameters (Ejection Fraction, NT-proBNP, HF severity index, NYHA index, etc.). The cardiologists performed a blinded HF assessment by evaluating the specific clinical parameters only.

The cardiologists’ classification represented the external clinical standard against which we checked the accuracy and predictive values of the *CARPEDIEM* algorithm.

In the following sections, we describe the result of each step of this process.

## Results

### Development and Evaluation of the Preliminary Inference Rules

To build inference rules, it is critical to determine what elements might be informative for identifying health conditions.

There are a variety of approaches for classifying patients into a particular phenotype, and our hypothesis is that the process of phenotype identification is more efficient if considering many different information sources. So, we began with the available data sets, including a set of conditions (hospitalization, drug treatment, and exemptions) taken in inclusive disjunction (OR).

The use of preliminary rules allowed to identify a total of 37,210 HF cases, equal to a prevalence rate of 6.1% (area 1: 5.9%; area 2: 6.5%), as shown in Table 3.

**Table 3:** Annual percentage prevalence rate of HF estimated either by GPs or referring to the preliminary and final rules (2011–2014).

Source	Area 1	Area 2	Area 1 + area 2
GPs assessment (GPstd)	1.4%	1.2%	1.3%
IC 95% lower	1.3%	1.1%	1.3%
IC 95% upper	1.5%	1.3%	1.4%
Preliminary rules (HFprR)	5.9%	6.5%	6.1%
IC 95% lower	5.8%	6.3%	6.0%
IC 95% upper	5.9%	6.6%	6.1%
CARPEDIEM algorithm	2.2%	2.5%	2.3%
IC 95% lower	2.2%	2.5%	2.3%
IC 95% upper	2.3%	2.6%	2.4%

These rates were four times higher than the value estimated by GPs (patients with life course documented clinical evidence), and this suggested a potentially high number of false positives influencing the accuracy of the rule-based system.

### Selection and Evaluation of the *CARPEDIEM* Algorithm

The broader definition of HF, referring to the inclusive disjunction (OR) among codes derived from different data sources, tended to overestimate the number of false-positive cases.

The narrower definition included in the final rules was more accurate in identifying HF cases. The prevalence rate estimated by running the *CARPEDIEM* algorithm was 2.3% (area 1: 2.2%; area 2: 2.5%), a value slightly higher than the GPs standard (Table 3).

### External Validation of the *CARPEDIEM* Algorithm

The patient group used for the external validation included 312 HF cases identified through the *CARPEDIEM* algorithm in the period 2011–2014 and 77 subjects not affected by HF.

Age and gender distribution of the patients in the validation subpopulation was different compared to the general population. In particular, individuals aged 65 years and older and males were overrepresented in the subpopulation compared to the reference cohort (76.3 vs 27.5% and 51.9 vs 47.5%, respectively).

This could be expected as the subpopulation used for the validation and composed of individuals who interacted with a public entity of the Regional Health Service (GM Foundation), which is particularly involved in managing patients with more severe cardiovascular diseases.

**Figure 2:**
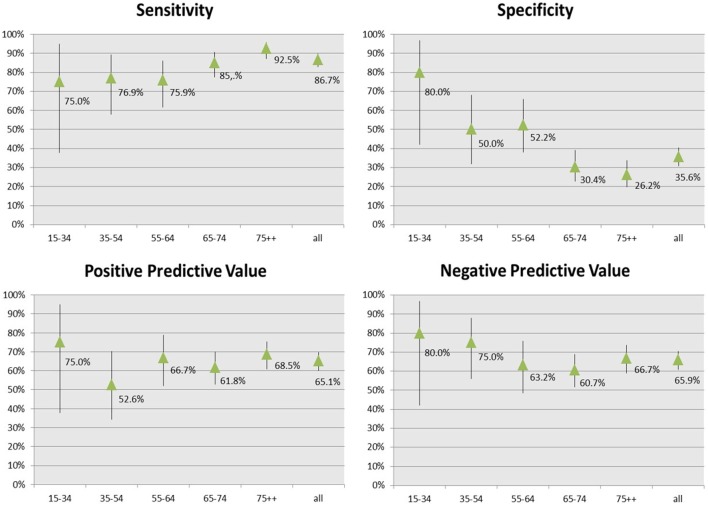
Accuracy and predictive measures of the *CARPEDIEM* algorithm.

Figure 2 shows the accuracy and predictive measures of the final rules estimated by the comparison with the external clinical standard.

The comparison with the clinical standard showed that the *CARPEDIEM* algorithm validity varied by age, with sensitivity that increased from 75 to 92% according to the increase of age, while specificity sharply decreased.

Positive predictive value (PPV) and negative predictive value (NPV) were generally higher than 65% and varied by age.

The *CARPEDIEM* algorithm validity also varied according to the worsening of HF severity estimated by using NYHA and HF severity index (Tables 4 and 5).

**Table 4:** Degree of concordance by NHYA score between *CARPEDIEM* algorithm and external standard.

NYHA score	Cohen’s Kappa	Strength of agreement	*CARPEDIEM* algorithm, number of cases (number of patients)^a^
I—Cardiac disease, but no symptoms and no limitation	0.15	Poor	17 (22)
II—Mild symptoms and slight limitation during ordinary activity	0.57*	Moderate	84 (94)
III—Marked limitation in activity due to symptoms, even during less than ordinary activity	0.57*	Moderate	85 (98)
IV—Severe limitations	0.67*	Good	14 (16)

*^a^Limited to 200 patients with heart failure clinically validated and New York Heart Association score ≥1*.

*p < 0.05.

**Table 5:** Degree of concordance by heart failure (HF) severity score between the *CARPEDIEM* algorithm and the external standard.

Heart failure severity score	Cohen’s Kappa	Strength of agreement	*CARPEDIEM* algorithm, number of cases (number of patients)^a^
1	0.44*	Moderate	98 (121)
2	0.66*	Good	28 (29)
3	0.70*	Good	70 (76)

*^a^Limited to 226 patients with HF clinically validated and New York Heart Association (NYHA) score ≥1*.

*p < 0.05.

### Validation of the *CARPEDIEM* Algorithm against GPs Assessment

Table 6 shows the accuracy and predictive measures of the *CARPEDIEM* algorithm estimated by the comparison with true positive and negative HF cases as identified by GPs within the whole reference cohort (153,393 subjects).

**Table 6:** Accuracy and predictive measures of the *CARPEDIEM* algorithm, estimated by the comparison with the GPs assessment (GPstd) by area.

	Sensitivity IC 95%	Specificity IC 95%	PPV IC 95%	NPV IC 95%	Likelihood ratio (LR) (+) IC 95%	LR (−) IC 95%
Area 1	72.5	93.8	14.3	99.6	11.7	0.3
	72.2	93.6	14.1	99.5	11.3	0.3
	72.7	94.0	14.5	99.6	12.1	0.3

Area 2	83.3	92.7	12.5	99.8	11.5	0.2
	83.0	92.5	12.3	99.7	11.1	0.2
	83.6	92.9	12.8	99.8	11.8	0.2

*CARPEDIEM* algorithm performance is also expressed in terms of LRs, the likelihood of a given test result in a person with the disease, compared with the likelihood of this result in a person without the disease (15). In other words, LRs indicate how much a given test result will raise or lower the probability of having the event that the test is designed to predict (16).

Predictive values give probabilities of abnormality for particular test results, but they depend on the prevalence of abnormality in the study sample and they can be rarely generalized beyond the study, except when the study is based on a suitable random sample, as is sometimes the case for population screening studies (17).

Otherwise, LRs are alternative statistics for summarizing diagnostic accuracy that have several particularly powerful properties making them more useful clinically than other statistics.

LR is portable, while predictive values of test are driven by the prevalence of the disease. Moreover, while predictive values infer test characteristics to the population, LRs can be applied to a specific patient (18) and again the LR is thought to be more stable because sensitivity and specificity usually vary in opposite directions (19).

As with all ratios, LRs range from zero to infinity and hence the further LR is from 1, the greater effect is on the probability of disease. In particular, tests with LR(+) between 2 and 5 are only somewhat useful, between 5 and 10 are moderately useful, tests with LR less than 2 are not useful, and tests with LR(+) greater than 10 are considered very useful.

LR(−) is the proportion between false- and true-negative patients. Tests with LR(−) between 0.2 and 0.5 are only somewhat useful, between 0.1 and 0.2 are moderately useful, tests with LR greater than 0.5 are of no use, and tests with LR(−) less than 0.1 are considered very useful.

In both areas, LR(+) values showed the very useful results of applying the *CARPEDIEM* algorithm in raising the probability of having HF, while its ability in correctly identifying the true negatives is moderate (Table 6).

**Figure 3:**
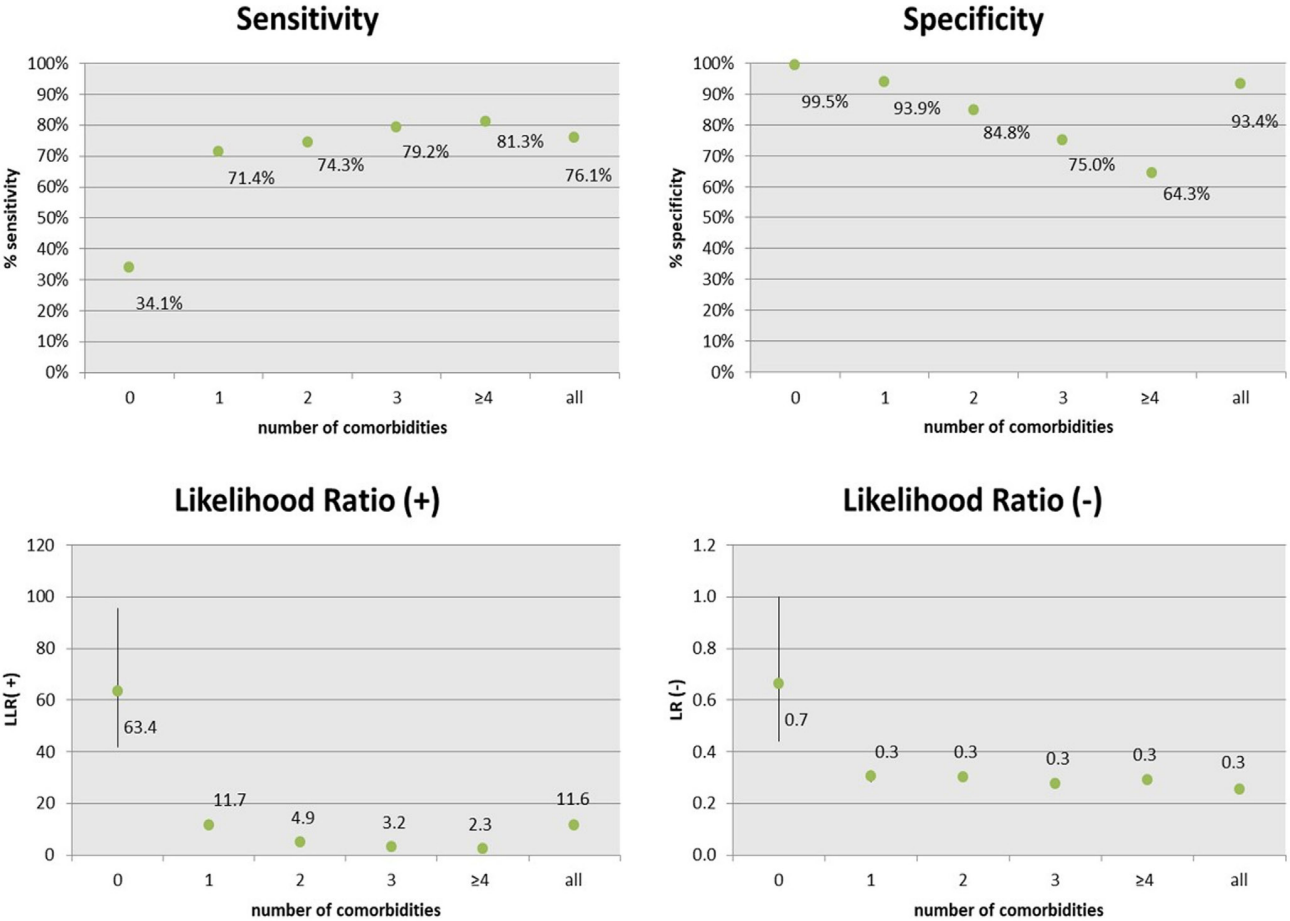
Accuracy and predictive measures of the *CARPEDIEM* algorithm, estimated by the comparison with the general practitioners assessment (GPstd), by the number of comorbidities.

In term of accuracy, the *CARPEDIEM* algorithm showed a good sensitivity and a very high specificity (Figure 3), but these rates varied according to the number of different pathological categories the patient belongs to, or in other term, according to the complexity of the clinical status (severity). As a general rule, sensitivity increased mainly depending on two types of evidence: (1) among clinically similar or related diseases many medications overlap and (2) patients with a higher number of comorbidities have more probability of being hospitalized and this makes these patients better identifiable.

## Discussion

Heart failure happens when the heart cannot pump enough blood and oxygen to support other organs in the body. It is a complex condition that can result from coronary artery disease, heart attack, cardiomyopathy, high blood pressure, diabetes, thyroid disease, kidney disease, valve disease, etc. and requires a specialist for both accurate diagnosis and therapeutic intervention. Globally it remains the single largest cause of death. The prevalence of this condition in Italy is about 1.25% [95% confidence interval (CI), 1.23–1.27], and the incidence rate was 1.99 per 1000 person-years (95% CI, 1.81–2.08) that is expected to grow (20).

As with most chronic diseases, in the absence of effective prevention, early detection and appropriate treatment are critical for patient management and quality of life.

The estimated cost for HF in Italy is $39.2 billion annually (21).

Because of the variability in cause, in presentation, in patterns of comorbidities, etc. and especially in the differences in presentation of symptoms between males and females, it is critical that patients be identified who are at risk or in early stages of this disease so that proper diagnosis and treatment can be obtained. The complexity associated with HF makes it significantly susceptible to the errors of misdiagnosis and missed diagnosis, especially in patients who are not engaged with a cardiac specialist.

**Figure 4:**
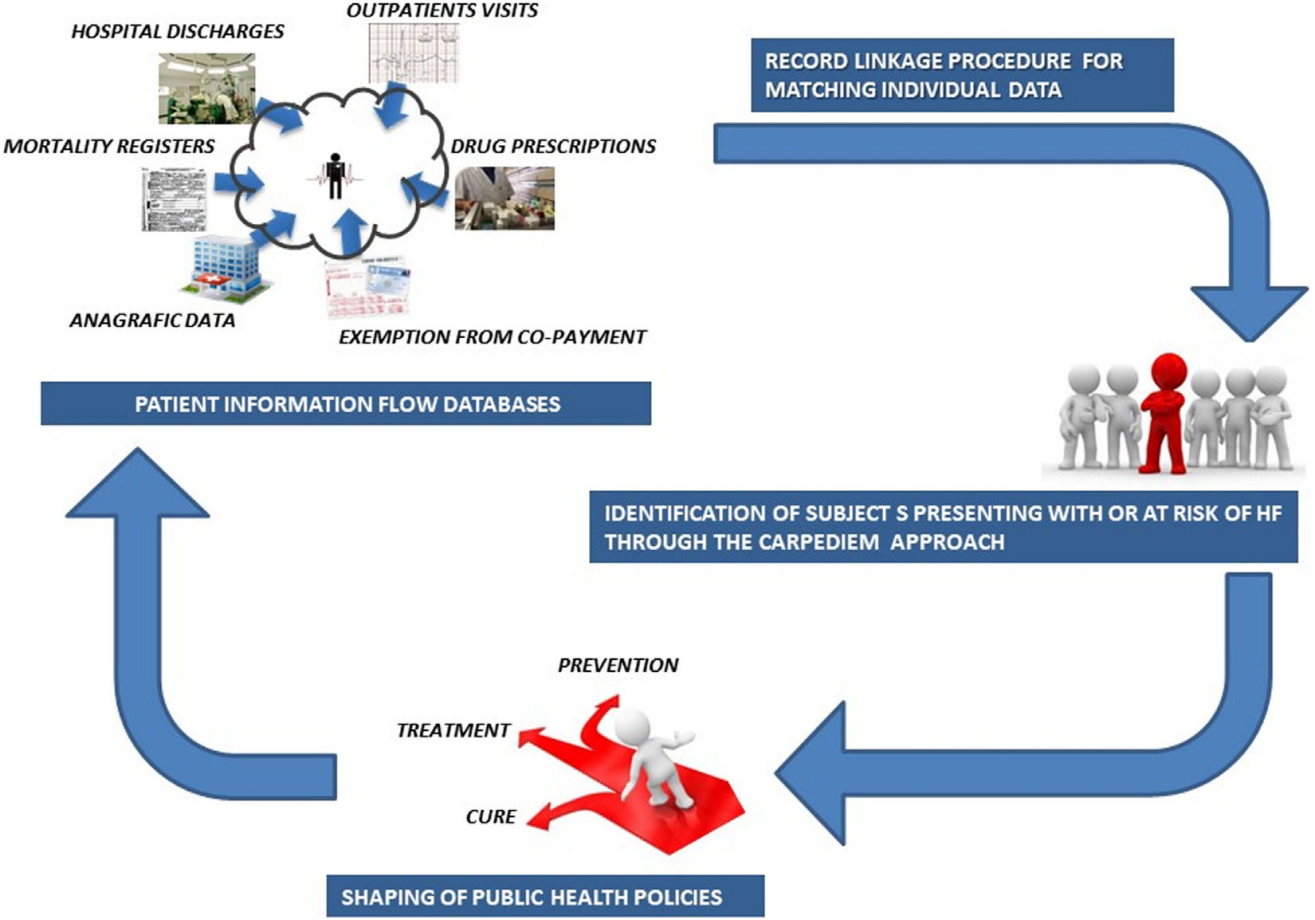
Schema of the *CARPEDIEM* approach.

Understanding the complexity associated with the healthcare process is vital for achieving scalable and high-throughput phenotyping algorithms that might operate on the administrative data contained in the NHS data repositories (Figure 4).

A significant aspect of the *CARPEDIEM* approach is that it is based on the data that exist with patient information flow databases and is not reliant on the electronic health record to accomplish its goals.

The nature of HF, not considered to be a discrete condition, but a “complex clinical syndrome” (7, 22) characterized by high comorbidity burdens, makes the integration among different healthcare process events mandatory in defining HF cases.

Moreover, recent literature stated that most if not all age-related diseases share low-grade chronic inflammation termed “Inflammaging,” which is a highly significant risk factor for both morbidity and mortality in elderly people (23).

Chronic inflammation can be prevented and cured (24), but there is an urgent need to better understand the links between molecular features and phenotype to develop strategies to deal with their implications for the individual patient (25).

Currently used organizational structure of the taxonomy of diseases, not taking into account that multiple different diseases share a common molecular cause, is so rigid as to preclude description of the complex interrelationships that link diseases to each other and to the vast array of causative factors.

This confirms the hypothesis that combining different variables from many sources of administrative data (hospitalization, drugs, ambulatory care record) is the best strategy in identifying cases of specific disease basing on their association with specific healthcare process events (10).

For instance, while HF may contribute to the need of hospitalization caused by other health problems, the HF diagnosis may not be entered on the discharge record. Considering diagnosis codes from hospital discharge as a sole criterion to identify cases of HF, leads to underestimate the prevalence of HF (26).

In integrating different sources of data, it is mandatory to consider that the inclusion of drug prescription codes in the rule-based identification system could increase the HF false-positive rate, because among clinically similar or related diseases, many medications overlap (27). Otherwise it is also true that the pharmacological treatment of patients with chronic HF seems to be acceptably adherent to the recommendations (28).

In this study, we specify, apply, and validate the most suitable algorithm to identify those patients with or at risk to develop HF and we set this as the first step for improving the Precision Public Health approach.

In particular, we tested two different combinations of diagnostic and therapeutic codes used for identifying individuals with HF from the administrative data of the general population.

The broader combination was based on the inclusive disjunction (OR connective) among drug, diagnoses, and exemption codes, while in the narrow combination, the *CARPEDIEM* algorithm, drug codes were grouped in some possible therapeutic strategies for HF. These subgroups were taken in inclusive disjunction (OR connective) with diagnoses and exemption codes.

The HF prevalence rate obtained by running the broader combination is four times higher than the value estimated by GPs, while the prevalence from the *CARPEDIEM* algorithm is only slightly higher (2.3%; 2.3–2.4%).

The accuracy and the predictive values for the *CARPEDIEM* algorithm with respect to the GPs and the clinical standards are highly consistent with those from other studies in terms of validity of HF diagnoses in administrative databases. In particular, the sensitivity of the *CARPEDIEM* algorithm compared to the clinical standard ranges from 72 to 92% according to the increase of age, with an average value of 88%, which is consistent with those values estimated among the high-quality studies selected by McCormick et al. (29).

The sensitivity of the *CARPEDIEM* algorithm compared to the GPs standard is a bit lower (76.1%; IC 95%: 75.3–76.2), but it varies from 34.1 to 81.3% according to the complexity of the clinical status, expressed as the number of comorbidities belonging to different categories of disease (disability, psychiatric disorders, chronic renal insufficiency, transplantation, neoplasms, cardiovascular diseases, chronic obstructive pulmonary disease, gastroenteropathy, neuropathy, autoimmune, endocrine and metabolic diseases, diabetes, rare diseases, pregnancy, and other health conditions that cannot be classified as chronic disease or pregnancy).

Evidence of the relationship among clinical status severity and the *CARPEDIEM* algorithm validity also comes from the extent of agreement with the clinical scores assessed within the validation cohort (NHYA and HF severity), which increases from less severe conditions to the worst (Cohen’s Kappa from 0.44 to 0.70).

These results suggested that severe cases of HF may be recorded more often in administrative databases than mild severity ones.

PPV is moderate (65.1%) when the final rules have been compared to the clinical standard and very low when compared to the GPs standard (13.6%; IC 95%: 13.4–13.8). On the contrary, the best NPV refers to the GPs standard (99 vs 65%).

This means that the rule-based system is more consistent with a clinical evaluation performed in a specialist setting while it has a more intense ability in ruling out the false negative HF cases within a more general clinical setting (General Medicine). This could be critical to add in the early detection of patients either at risk for HF or in early stages of HF and enable their referral to specialists who can further optimize their cardiovascular care.

This could reflect that PPV and NPV are both dependent on the prevalence of the condition in the reference population. As a consequence, PPV will be higher in a specialist setting where the prevalence is higher, while the NPV will be better in a general setting were the prevalence is lower.

Further support about the rule-based system validity respect to the general setting was provided by the LR values estimated through the comparison with GPs standard. In particular, LR (+) is greater than 10 in both geographic areas, and this means that an individual classified for HF by the *CARPEDIEM* algorithm is more than 10 times likely to have had a HF diagnosed by GPs with respect to a not classified individual.

In summary, *CARPEDIEM* establishes the potential that an algorithmic approach, based on integrating the administrative data with other public data sources, can present the opportunity to develop low-cost and high-value population-based evaluations for improving public health and impacting public health policies. This suggests that the *CARPEDIEM* approach can become a significant contributor to the ultimate goal of Personalized Medicine by providing a reliable and reproducible component of disease diagnosis. Moreover, the core of the algorithm can be easily customized to process data sources other than those we used in our study, provided that they contain standardized clinical information. This represents a potential for evolution and improvement of predictive levels of the *CARPEDIEM* algorithm not only in the heart diseases.

Further research will be carried out by using alternative methodologies such as the Social Network Analysis, a specific tool for enhancing hypothesis generation and with a particular ability in identifying complex and hidden relationships among the data, exposing them for further appropriate statistical verification and supporting complex investigation about undiagnosed HF and gender differences in the clinical presentation of HF.

Thus, the ability to examine readily accessible data, such as the administrative data contained in the National Health Service database, can be a critical component of a broad population-focused surveillance program in HF and also lead to its potential application in other chronic and complex disorders, and *CARPEDIEM* provides an initial prototype for such development.

## Ethics Statement

This study was carried out in accordance with Italian Legislative Decree 196/2003 on privacy, as stated by the ethical committee of “Area Vasta Nord Ovest PISA” (approval document no. 539/2015, on February 20, 2015). Written informed consent was given by the patients for their information to be stored in the databases used in this study. Written consent follows the criteria established in the Arts. 13 and 22 of the Italian Legislative Decree 196/2003 on privacy. GPs collect written consent only once, at the time of taking in charge of each patient. GPs usually store written consents in their digital archives. Written consent is mandatory whenever patient and NHS interact. All the written consents also regards statistical processing of the data for research purposes. In this case, data must be anonymous, and the patient must not be identifiable in any way. The linkage procedure among the databases was performed using encrypted unique identifier codes created for each patient by the Local Administrative Authority, which is the health provider legally authorized for the processing of sensitive data.

## Author Contributions

MF provided substantial contributions to the epidemiological conception and design of the work, assisted with acquisition of data and algorithm identification, completed all analysis, led interpretation of data, drafted the work, and revised it critically. SP provided relevant technical contributions to the design of the work, assisted with acquisition of data and algorithm identification, led interpretation of data, and assisted in drafting and revising the work. CP and ME provided substantial contribution to the clinical conception of the work and algorithm identification, acquired and provided clinical data, and participated to the final approval of the version to be published and ensured that questions related to the clinical accuracy or integrity of any part of the work are appropriately investigated and resolved. SM assisted in designing the work, drafting the paper, and revising it critically. SM also provided the final approval of the version to be published and ensured that questions related to the accuracy or integrity of any part of the work are appropriately investigated and resolved.

## Conflict of Interest Statement

The authors declare that the research was conducted in the absence of any commercial or financial relationships that could be construed as a potential conflict of interest.
